# Fleas and trypanosomes of peridomestic small mammals in sub-Saharan Mali

**DOI:** 10.1186/s13071-016-1818-5

**Published:** 2016-10-11

**Authors:** Tom G. Schwan, Job E. Lopez, David Safronetz, Jennifer M. Anderson, Robert J. Fischer, Ousmane Maïga, Nafomon Sogoba

**Affiliations:** 1 Laboratory of Zoonotic Pathogens, Rocky Mountain Laboratories, National Institute of Allergy and Infectious Diseases, Hamilton, MT USA; 2 Pediatric Tropical Medicine, Baylor College of Medicine, Houston, TX USA; 3 Laboratory of Virology, Rocky Mountain Laboratories, National Institute of Allergy and Infectious Diseases, Hamilton, MT USA; 4 Zoonotic Diseases and Special Pathogens, Public Health Agency of Canada, Winnipeg, MB Canada; 5 Laboratory of Malaria and Vector Research, National Institute of Allergy and Infectious Diseases, Twinbrook, MD USA; 6 International Center of Excellence in Research (ICER-Mali), Faculty of Medicine and Odontostomatology (FMOS), University of Sciences, Techniques and Technologies of Bamako, Bamako, Mali

**Keywords:** Siphonaptera, Rodents, Shrews, Trypanosomosis, West Africa

## Abstract

**Background:**

Fleas are obligate blood-feeding ectoparasites and vectors of several bacterial zoonotic pathogens as well as trypanosomes that parasitize rodents and other small mammals. During investigations of tick- and rodent-borne diseases in Mali, West Africa, we included fleas and rodent-borne trypanosomes, both of which are poorly known in this country, but are attracting greater public health interest.

**Methods:**

Small mammals were captured in 20 Malian villages from December 2007 to October 2011. Fleas were collected and identified to species, and thin blood smears were prepared, stained and examined microscopically for trypanosomes.

**Results:**

We captured 744 small mammals, 68 (9.1 %) of which yielded fleas. Two species of fleas, *Xenopsylla cheopis* and *Xenopsylla nubica*, were collected from six species of rodents and one species of shrew. Multimammate rats, *Mastomys natalensis*, were hosts for 58.5 % of all fleas collected. *Xenopsylla cheopis* was found in the moister southern savannah while *X. nubica* was mostly restricted to the drier Sahel. Trypanosomes were found in 3 % of 724 blood smears, although 91 % of parasitemic animals originated from two villages where black rats (*Rattus rattus*) and *M. natalensis* were the primary hosts and *X. cheopis* the dominant flea. The trypanosomes were morphologically consistent with the *Trypanosoma* (*Herpetosoma*) *lewisi* group, flea-borne hemoflagellates that parasitize domestic rats.

**Conclusions:**

*Xenopsylla cheopis* and trypanosomes parasitize peridomestic rats that commingle with people in southern Mali. Given the increasing awareness of flea-borne trypanosomes as possible human pathogens, we hope our findings will stimulate future investigators to examine the potential public health significance of flea-borne trypanosomosis in West Africa.

**Electronic supplementary material:**

The online version of this article (doi:10.1186/s13071-016-1818-5) contains supplementary material, which is available to authorized users.

## Background

Fleas (Insecta: Siphonaptera) are obligate blood-feeding ectoparasites as adults and serve as vectors of several bacterial zoonotic pathogens, the most devastating being *Yersinia pestis*, the causative agent of plague [1]. Additionally, many species of fleas are biological vectors of trypanosomes, which cycle between these wingless insects and a diversity of wild and peridomestic small mammals in many parts of the world [2, 3]. The diversity of fleas and mammals parasitized by these blood-borne flagellates suggests a long evolutionary history [4]. Historically considered nonpathogenic in humans, there is an increasing awareness that these rodent-borne parasites have the potential to cause disease in people [5, 6].

During a field investigation of tick-borne relapsing fever in Mali [7], we live-trapped small mammals across the southern region of the country, collected fleas, and prepared thin blood smears from the animals. Here we show that the Oriental rat flea, *Xenpsylla cheopis*, one of the most important urban vectors of the plague bacterium, *Y. pestis*, and the agent of murine typhus, *Rickettsia typhi* [8], is widespread in southern Mali. We also show that flea-borne trypanosomes of possible public health significance parasitized peridomestic rodents in several villages where the presumed flea vector was also found.

## Methods

### Small mammals and fleas

The procedures used to capture, handle, and identify small mammals were described in detail previously [7]. In summary, 20 villages across southern Mali were visited on 27 occasions from December 2007 to October 2011. The villages were located in the moist savannah in the south, to the drier savannah and Sahel regions to the northeast. Animals were captured alive in Sherman live traps (H.B. Sherman Traps, Tallahassee, FL, USA) and euthanized in plastic bags by inhalation of Isoflurane (Fluriso, Vet ONE, MWI Veterinary Supply, Boise ID, USA). Blood samples were collected and thin smears were made on glass microscope slides. Ear punch biopsies were taken for DNA extraction and determination of the mitochondrial (mt) cytochrome-*b* (*cyt-b*) sequence to aid in species identification as described [7], and skulls were prepared from representative species. Each animal was brushed lightly over a white enamel pan to dislodge fleas from their hair. Those specimens and fleas that abandoned the host in the clear plastic anesthesia bag were collected in 70 % ethanol for subsequent examination.

### Identification of fleas

Fleas preserved in ethanol were sequentially rehydrated in water, soft tissues digested in 10 % KOH, rinsed in water, dehydrated in increasing concentrations of ethanol (25, 50, 75 and 100 %), cleared in xylene, and mounted in Canada balsam on glass microscope slides. The mounted specimens were examined with a Nikon Eclipse E800 bright-field microscope (Nikon Instruments Inc., Melville, NY, USA) and fleas were identified to species with published keys and illustrations [9] and comparisons to reference material in the first author’s (TGS) collection.

### Examination of blood smears

Thin blood smears on glass microscope slides were fixed with 100 % methanol, stained with the QUICK III statpak kit (Astral Diagnostics Inc., West Deptford, NJ, USA), and 50 fields were examined for trypanosomes by bright-field microscopy at 600× with a 60× oil immersion lens. The mean number and range of trypanosomes counted are presented for one field of view, which had an area of 0.126 mm^2^. Images were taken with a Nikon Digital Camera DXM1200C and ACT-1C software package Version 1.0.1.5 and displayed on a flat-screen computer monitor. Total length of each parasite was determined by super-imposing by hand a thin pliable filament of solder (dia. = 0.81 mm) (Alpha Fry Technologies, Altoona, PA, USA) over the longitudinal axis of the trypomastigote from the apex of the posterior end to the terminus of the anterior free flagellum. When the solder matched the shape and length of the parasite, the curved filament was straightened, its length measured in mm, and then calibrated to μm by comparison to an image of a stage micrometer with 0.02 mm divisions.

## Results

We captured 744 small mammals that included 11 species of rodents and three species of shrews [7] (Additional file 1: Table S1). In all, 176 fleas were collected from 68 animals (9.1 % of captures) in 14 villages (Table 1), which included six species of rodents and one species of shrew (Table 2, Additional file 2: Table S2). DNA sequences of the mammalian mt *cyt-b* gene supported the identifications of the host species and are available in GenBank (accession numbers JX292860–JX292895). Voucher specimens comprised of 60 skulls also supported the identifications and are deposited in the Smithsonian Institution’s Collection of Mammals (accession numbers USNM 599343–USNM 599402).

**Table 1:** Malian villages, their administrative districts, and geographic coordinates where fleas were collected

Village	District	Latitude (North)	Longitude (West)
Djougounte	Kayes / I	14°07'19.2"	09°58'32.5"
Djidian	Kayes / I	13°12'02.5"	09°27'13.7"
Bozokin	Koulikoro / II	12°41'53.2"	08°14'35.2"
Kenieroba	Koulikoro / II	12°06'43.9"	08°19'55.9"
Fourda	Koulikoro / II	12°05'29.4"	08°20'06.0"
Doneguebougou	Koulikoro / II	12°48'18.0"	07°58'49.1"
Soromba	Sikasso / III	10°35'20.8"	07°09'20.5"
Belenikegny	Ségou / IV	13°22'56.6"	04°55'00.1"
Molibana	Mopti / V	14°00'55.8"	04°13'51.6"
Sama	Mopti / V	14°55'24.6"	03°53'49.9"
Sinkerma	Mopti / V	14°22'50.9"	03°34'05.5"
Petaka	Mopti / V	15°01'25.3"	02°50'55.0"
Kalibombo	Mopti / V	14°24'01.4"	03°36'01.8"
Doucombo	Mopti / V	14°21'18.7"	03°39'26.3"

**Table 2:** Species and numbers of small mammals yielding *Xenopsylla nubica* and *Xenopsylla cheopis* fleas in southern Mali

Host species (No. of infested specimens)	*X. nubica*	*X. cheopis*	Total
	♂/♀ (Total)	♂/♀ (Total)	
*Mastomys natalensis* (35)	4/19 (23)	38/42 (80)	103
*Mastomys erythroleucus* (6)	2/2 (4)	3/– (3)	7
*Praomys daltoni* (5)	1/4 (5)	2/2 (4)	9
*Arvicanthis niloticus* (4)	–/1 (1)	2/2 (4)	5
*Taterillus gracilis* (5)	3/6 (9)	–/–	9
*Rattus rattus* (2)	–/–	3/6 (9)	9
*Crocidura olivieri* (11)	1/2 (3)	16/15 (31)	34
Total (68)	11/34 (45)	64/67 (131)	176

**Fig. 1:**
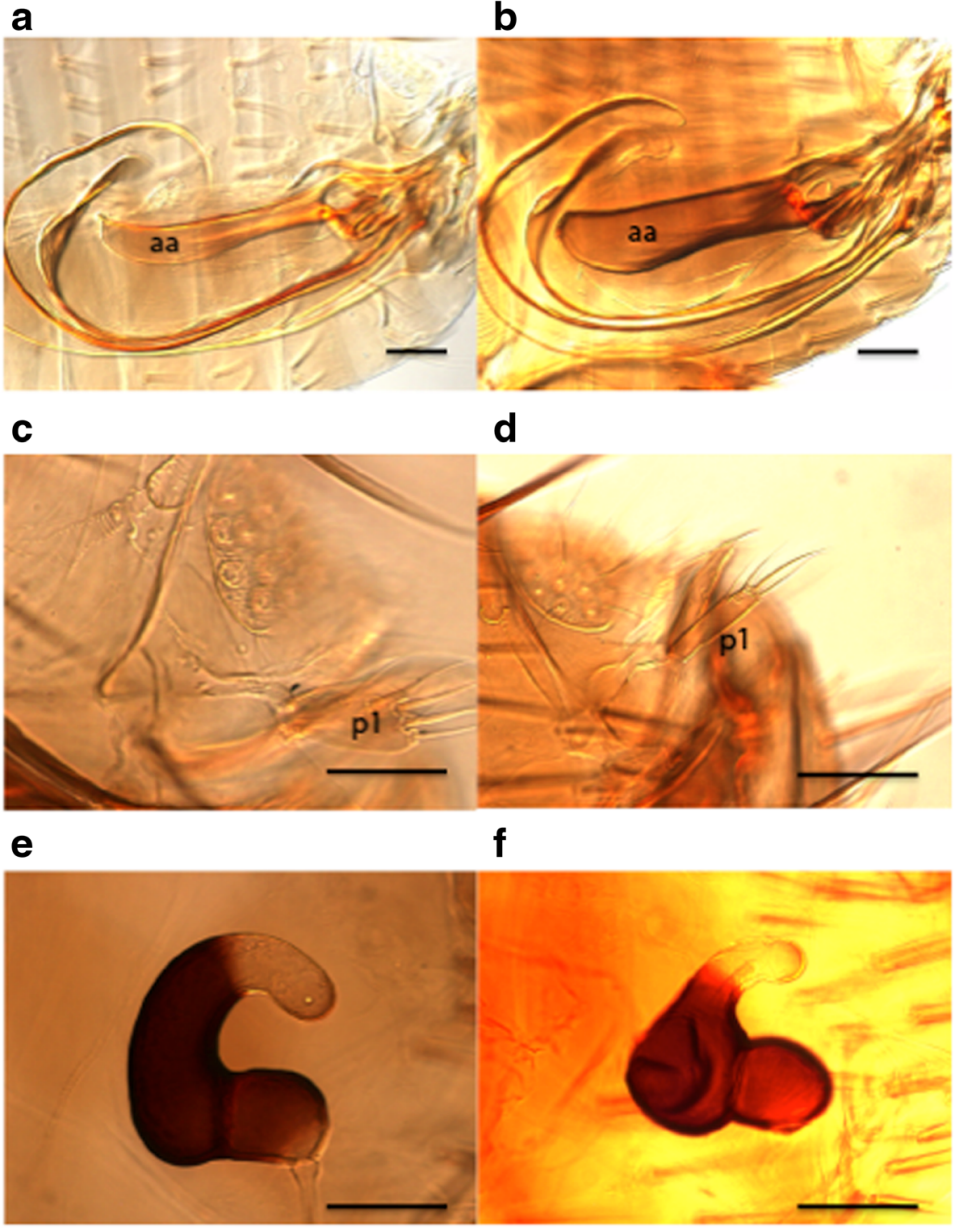
Morphological characters for identification of *Xenopsylla cheopis* (**a**, **c**, **e**), and *Xenopsylla nubica* (**b**, **d**, **f**). **a**, **b** Aedeagal apodeme of male (aa). **c**, **d** First process of male clasper (p1). **e**, **f** Spermatheca of female. *Scale-bars*: 0.1 mm

Only two species of fleas, *Xenopsylla cheopis* and *Xenopsylla nubica*, were collected from the hosts and villages sampled (Table 2) and both species were represented by adequate numbers of males and females to support their identification. Multiple characters of the genitalia including the shape of the aedeagal apodeme (penis-plate) and first process (p1) of the clasper in the males and the spermatheca in the females, visible in the cleared and mounted specimens (Fig. 1), were used to identify the species. Multimammate rats, *Mastomys natalensis*, comprised approximately half (51.5 %) of the animals that yielded fleas and were hosts for 58.5 % of all fleas collected. Both species of fleas were found on five of the seven host species infested (Table 2). The gerbil *Taterillus gracillus* yielded only *X. nubica* and the black rat *Rattus rattus* was infested with only *X. cheopis*.

**Fig. 2:**
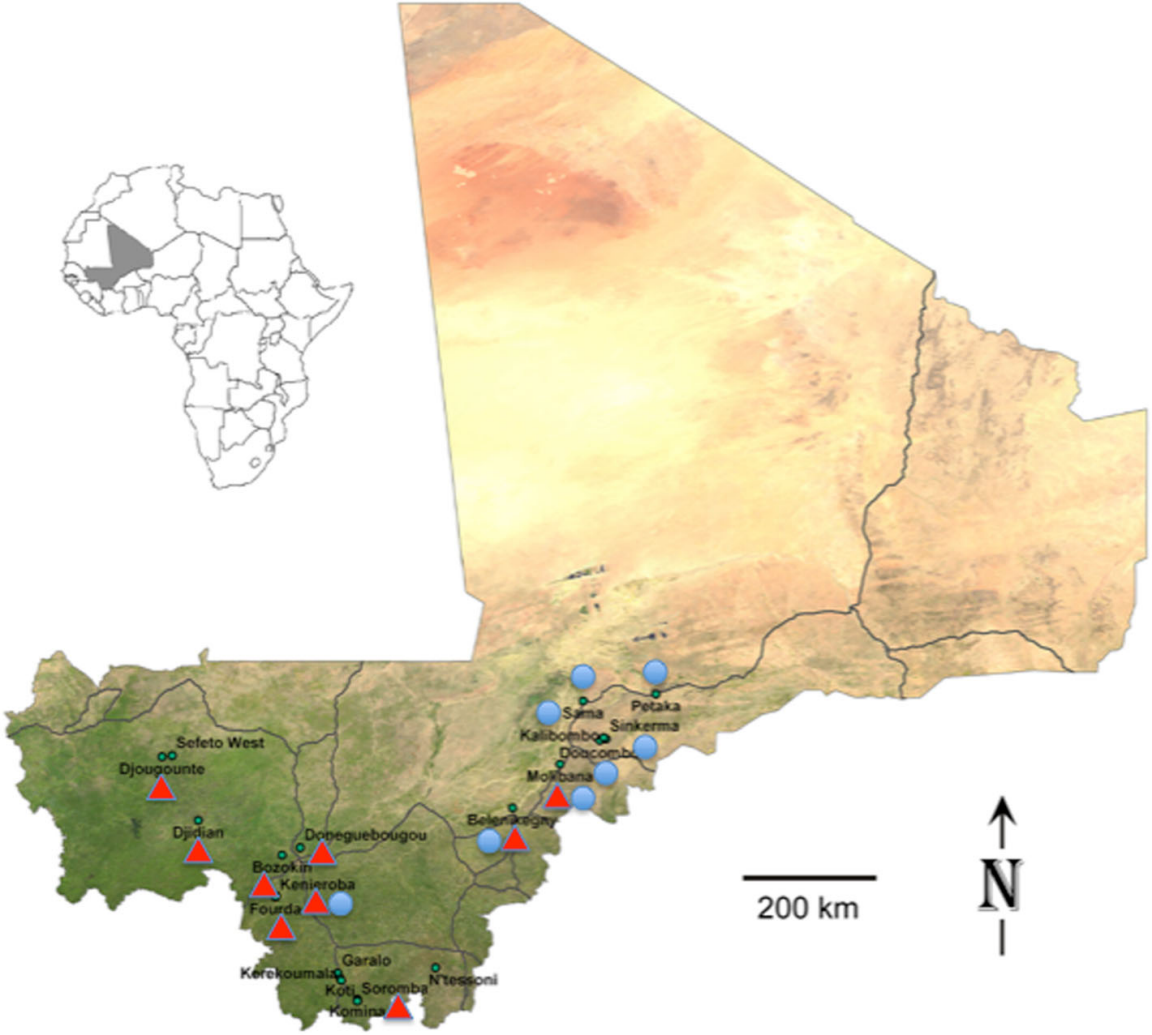
Distribution of collection sites for *Xenopsylla cheopis* (*red triangles*) and *Xenopsylla nubica* (*blue circles*) in southern Mali

Both species of fleas were widespread, being found in many villages, however they displayed regional differences in distribution with little geographical overlap (Fig. 2). *Xenopsylla cheopis* was collected primarily in the moister southern savannah while *X. nubica* was found in the drier Sahel to the northwest. We collected both species in only three villages, and just one of the 68 animals yielding fleas was infested with both species, a *Mastomys erythroleucus* trapped in Molibana, located in a transitional zone between the savannah and Sahel.

Trypanosomes were observed microscopically in 22 of 724 stained blood smears from three of 14 mammal species captured, *R. rattus*, *M. natalensis* and *M. erythroleucus* (Table 3). While the overall prevalence of positive smears was low at only 3 %, 20 of the 22 (91 %) infected animals came from just two of the 20 villages, Belenikegny and Doneguebougou. In Belenikegny, 53.8 % of the *R. rattus* (14 of 26 captured) were parasitemic, and infected black rats were found during all three visits to this village (Table 3). In Doneguebougou, 10.5 % of the *M. natalensis* (4 of 38 captured) were infected during our first visit but none of the 41 animals was infected during our second visit. The levels of parasitemia varied considerably among the infected animals, from only one trypanosome seen in 50 microscopic fields, to averages of 30 or more in one field (Table 3). The infected *R. rattus* in Belenikegny had mean parasitemias ranging from 1.5 to nearly 10 trypanosomes per field. *Xenopsylla cheopis*, the probable vector for these trypanosomes, was found on these host species in both villages.

**Table 3:** Malian villages, dates, host species and trypanosome numbers in thin blood smears

Village	Date	Host species	Positive/Total	% Positive	Mean parasite load per field (range)
Belenikegny	January 2009	*R. rattus*	3/6	50.0	1.54 (0.02–3.30)
Belenikegny	January 2010	*R. rattus*	7/13	53.8	7.34 (0.02–26.40)
Belenikegny	September 2010	*R. rattus*	4/7	57.1	9.91 (0.96–19.60)
Belenikegny	January 2009	*M. erythroleucus*	1/11	9.1	24.80
Doneguebougou	June 2009	*M. natalensis*	4/38	10.5	2.42 (0.50–5.20)
Doneguebougou	June 2009	*M. erythroleucus*	1/2	50.0	35.00
Doneguebougou	September 2011	*M. natalensis*	0/41	0	0
N’Tessoni	June 2009	*M. natalensis*	1/15	6.7	3.00
Soromba	June 2009	*M. natalensis*	1/25	4.0	33.80

**Fig. 3:**
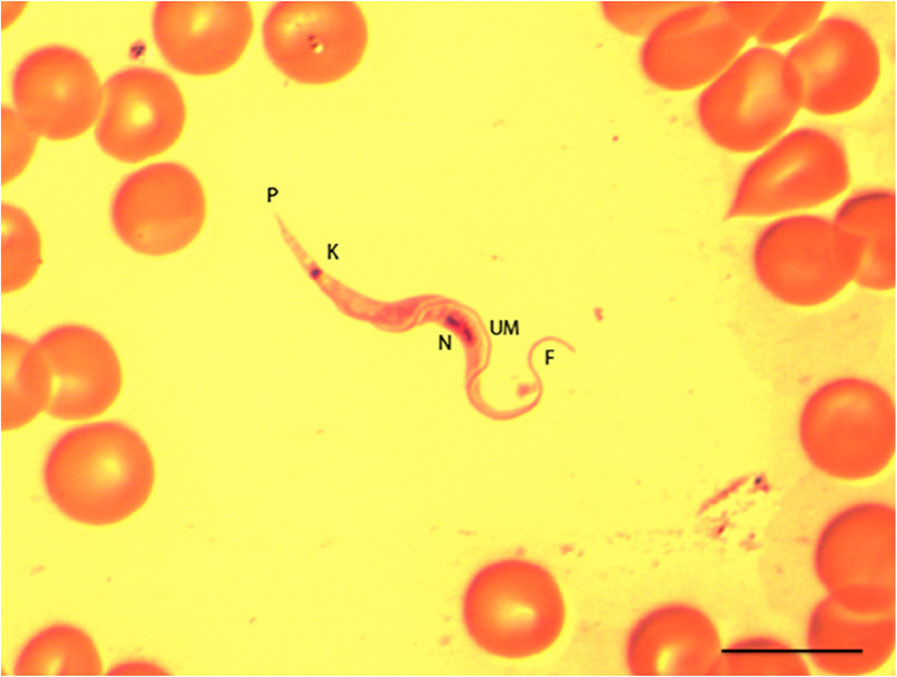
*Trypanosoma lewisi* /*T. lewisi*-like trypomastigote in the blood of *Rattus rattus*, Belenikegny, Mali. *Abbreviations*: P, posterior margin; K, kinetoplast; N, nucleus; UM, undulating membrane; F, flagella. *Scale-bar*: 10 μm

The trypanosomes were observed in fixed, stained blood smears and not identified by molecular methods. However, their gross morphological characters were consistent with the *Trypanosoma* (*Herpetosoma*) *lewisi* group, parasites of rodents that are transmitted by fleas, by having an acutely pointed posterior end, a large subterminal kinetoplast, oval nucleus anterior to the kinetoplast, large undulating membrane, and free anterior flagellum [3, 6] (Fig. 3). The total length was determined for 25 trypomastigotes in the blood smear of one *R. rattus* captured in Belenikegny. The mean length was 33.0 μm (range 31.0–33.5 μm), which is consistent for trypanosomes in the subgenus *Herpetosoma* and the *T. lewisi* group [3, 6]. Because the fleas were cleared and permanently mounted in Canada balsam for identification, their analysis for trypanosome infection was not possible.

## Discussion

The flea faunas are well known in many parts of the world, especially in regions endemic for plague, which is the most significant bacterial disease associated with the bites of these infected blood-feeding ectoparasites [10]. However, those regions historically free of plague during the third pandemic of the late 19th and 20th centuries have received less attention regarding the diversity, distribution and seasonal abundance of fleas. Mali, a large (1,240,192 km^2^) landlocked country in West Africa, is apparently one such place, historically free of plague and the flea fauna poorly known [11, 12].

The low diversity of flea species we found was surprising given the wide geographic coverage we sampled and the diversity of small mammals we captured [13]. However, our sampling was restricted to peridomestic settings with 70 % of all captures being made inside houses and associated structures, while the remaining animals were captured immediately outside or in nearby cultivated gardens [7]. Thus our finding of 68 % of the animals that yielded fleas were captured indoors was a direct reflection of our total capture effort and trapping strategy. The low diversity of fleas was likely influenced by sampling only areas occupied and modified by humans.

Rothschild described and named both *X. cheopis* and *X. nubica,* the two species we found, based on specimens collected from rodents captured near Shendi, Sudan, in 1901 [14]. *Xenopsylla cheopis* has a nearly worldwide distribution due to its primary association with commensal rats that include the Norway (brown) rat, *Rattus norvegicus,* and the black (roof) rat, *R. rattus* [9, 15]. This flea is widely distributed in Africa and has an East African subspecies that parasitizes small mammals in both sylvan and domestic habitats [9, 16, 17]. While *X. cheopis* has been found in many West and North African countries [9, 18–20] including Senegal immediately to the west [11], we found no records of its occurrence in Mali. Beaucornu’s comprehensive review of the fleas in sub-Saharan Africa did not include *X. cheopis* or any other species of flea in the country [21].

*Xenopsylla nubica* has a much more limited distribution than that of *X. cheopis*, but is found in much of Africa and throughout the Middle East [9, 14, 15, 18–20, 22, 23]. This species lives in more desert and semi-desert environments and is most often found parasitizing various species of arid-dwelling rodents such as gerbils and jerboas [9, 15, 23]. Our collections of *X. nubica* reflected this preference for more arid environments, as most of our specimens came from the drier Sahel in the northwestern areas we sampled (Fig. 2). Our finding of this flea parasitizing domestic multimammate rats (*M. natalensis*) living inside human dwellings is unusual. As for *X. cheopis*, we were unable to find any previous documentation of *X. nubica* in Mali.

Bray [24] provided a comprehensive review of the earlier literature pertaining to parasitic protozoa of West Africa, which included records of *T. lewisi* parasiting *R. rattus* in Côte d’Ivoire, Ghana, Guinea and Nigeria. A century ago, Macfie [25] provided a detailed analysis of *T. lewisi* found in the blood on one *Epimys rattus* (= *R. rattus*) examined in Accra, Ghana, and Delanoë [26] described trypanosomes in numerous rodent species in Côte d’Ivoire, including *T. lewisi* infections in *Mus concha* (= *M. natalensis*). Not long ago, Dobigny and coworkers [27] found *T. lewisi* in *R. rattus* and the spiny mouse *Acomys johannis,* and *T. lewisi*-like trypanosomes in *M. natalensis* and *M. erythroleucus* in several villages in Niger, including Boumba, approximately 800 km to the east of Belenikegny. The trypanosomes were detected and identified by PCR and DNA sequence analysis of spleen tissues from the rodents. No blood smears were examined by microscopy, and no fleas were included in the study. Yet, this investigation is the most extensive work done on rodent-borne trypanosomes in West Africa and complements our findings of *T. lewisi*-like trypanosomes infecting peridomestic rats in nearby Mali.

*Trypanosoma lewisi* is primarily a parasite of *Rattus* species and has long been considered nonpathogenic in humans [3]. However, eight infections in people have been reported [28–34] (Table 4), and there is increasing interest in these rodent-borne protozoa as potential human pathogens [5, 6, 35]. Five of the patients described were only four months old or less, suggesting that infants may be more susceptible to infection.

**Table 4:** Reported cases of human trypanosomiasis with *Trypanosoma lewisi* / *T. lewisi*-like infections

Age	Gender	Country	Year	Reference
4 months	Male	Malaysia	1932	[28]
Adult	Male	India	1972	[29]
Adult	Female	India	1972	[29]
2 months	Female	Senegal^a^	2003	[30]
45 days	Male	Thailand	2005	[31]
7 weeks	Female	India	2006	[32]
57 years	Male^b^	India	2006	[33]
37 days	Male	India	2010	[34]

^a^Possible exposure in The Gambia

^b^Fatal case

Fleas are the biological vectors of *T. lewisi* and other rodent-borne trypanosomes [3]. *Xenopsylla cheopis* is an established vector for these parasites, and while not proven, *X. nubica* may also be a vector as are other species of *Xenopsylla* fleas [3]. While early experiments demonstrated that fleas can transmit these protozoa by *per os* during feeding [36], the consensus now is that *T. lewisi* and related parasites, which develop in the hindgut and rectum of fleas [37], are shed in flea feces. Rodents are infected by ingesting infected fleas or contaminated flea feces [3, 37, 38], but how those rare human patients became infected (Table 4) is unknown.

The southern-most regions of Mali remain endemic for human trypanosomiasis caused by *Trypanosoma brucei gambiensis* transmitted by tsetse flies (*Glossina* species), although the areas we sampled are now essentially free of this disease [39, 40]. During all of our travels and work across southern Mali [7, 41], we never encountered a single tsetse fly. Therefore, in those villages we sampled, trypanosome infections in humans may be caused by rodent- and flea-borne parasites. Infants living in rat and flea infested dwellings, such as we observed in several villages in southern Mali, might be at risk of infection with these parasites via the contamination of food. Exposure might also occur when contaminated items are accidentally placed in their mouths or rubbed against open skin wounds or mucous membranes.

## Conclusions

Two species of fleas, *X. cheopis* and *X. nubica*, and *T. lewisi*-like trypanosomes were found in southern Mali parasitizing peridomestic rodents. In those villages where black rats, rat fleas and trypanosomes commingle with human populations, flea-borne trypanosomiasis, especially in newborn infants, may be possible and worthy of future attention.

## Additional files

Additional file 1: Table S1. Malian villages investigated, dates, species and number of small animals captured. (DOCX 19 kb)
Additional file 2: Table S2. Date, location, host species, animal identification number, flea species, and number and sex collected. (DOC 125 kb)

## Abbreviations

aa: Aedeagal apodeme
mt *cyt-b*: Mitochondrial cytochrome-*b* gene
p1: First process of the clasper
