# Supplementary material for: Fleas and trypanosomes of peridomestic small mammals in sub-Saharan Mali
Source: Parasit Vectors. 2016 Oct 11;9:541. doi: 10.1186/s13071-016-1818-5 (PMC5057378; doi:10.1186/s13071-016-1818-5)
Supplement: Additional file 1: Table S1. — Malian villages investigated, dates, species and number of small animals captured. (DOCX 19 kb) [file 13071_2016_1818_MOESM1_ESM.docx]

**Additional file 1: Table S1.** Malian villages investigated, dates, species and number of small animals captured

| **Village** | **Date of collection** | **Species captured** | **Number captured** |
| --- | --- | --- | --- |
| Molibana | 6 December 2007 | *Mastomys natalensis* | 1 |
|  |  | *Mastomys erythroleucus* | 12 |
|  |  | *Praomys daltoni* | 3 |
|  |  | *Arvicanthis niloticus* | 7 |
|  |  | *Taterillus gracilis* | 1 |
| Sama | 7 December 2007 | *Arvicanthis niloticus* | 1 |
|  |  | *Taterillus gracilis* | 3 |
|  |  | *Crocidura viaria* | 1 |
|  |  | *Crocidura fulvastra* | 1 |
| Sefeto West | 9 December 2007 | *Praomys daltoni* | 2 |
|  |  | *Crocidura olivieri* | 1 |
| Djougounte | 10 December 2007 | *Mastomys erythroleucus* | 1 |
|  |  | *Praomys daltoni* | 3 |
|  |  | *Crocidura olivieri* | 3 |
|  |  | *Crocidura fulvastra* | 2 |
| Djidian | 11 December 2007 | *Mastomys natalensis* | 10 |
|  |  | *Praomys daltoni* | 8 |
|  |  | *Crocidura olivieri* | 1 |
| Bozokin | 12 January 2009 | *Mastomys natalensis* | 19 |
| Kenieroba | 13 January 2009 | *Mastomys natalensis* | 8 |
|  |  | *Mastomys erythroleucus* | 4 |
|  |  | *Crocidura olivieri* | 8 |
| Fourda | 14 January 2009 | *Mastomys natalensis* | 14 |
|  |  | *Crocidura olivieri* | 3 |
| Sinkerma | 17 January 2009 | *Mastomys natalensis* | 3 |
|  |  | *Praomys daltoni* | 7 |
|  |  | *Acomys airensis* | 4 |
|  |  | *Gerbillus campestris* | 1 |
|  |  | *Crocidura sp*. | 1 |
| Petaka | 18 January 2009 | *Mastomys natalensis* | 6 |
|  |  | *Mastomys erythroleucus* | 8 |
|  |  | *Praomys daltoni* | 16 |
|  |  | *Arvicanthis niloticus* | 4 |
|  |  | *Taterillus gracilis* | 2 |
|  |  | *Mus musculoides* | 1 |
|  |  | *Crocidura viaria* | 2 |
| Belenikegny | 19 January 2009 | *Mastomys natalensis* | 1 |
|  |  | *Mastomys erythroleucus* | 11 |
|  |  | *Mastomys huberti* | 3 |
|  |  | *Arvicanthis niloticus* | 1 |
|  |  | *Rattus rattus* | 6 |
|  |  | *Crocidura olivieri* | 5 |
| N’Tessoni | 5–6 June 2009 | *Mastomys natalensis* | 15 |
|  |  | *Mastomys erythroleucus* | 2 |
|  |  | *Praomys daltoni* | 1 |
|  |  | *Arvicanthis niloticus* | 1 |
|  |  | *Mus musculoides* | 3 |
|  |  | *Crocidura olivieri* | 3 |
| Soromba | 8 June 2009 | *Mastomys natalensis* | 25 |
| Doneguebougou | 12–14 June 2009 | *Mastomys natalensis* | 38 |
|  |  | *Mastomys erythroleucus* | 2 |
|  |  | *Praomys daltoni* | 1 |
|  |  | *Arvicanthis niloticus* | 1 |
|  |  | *Rattus rattus* | 1 |
|  |  | *Crocidura olivieri* | 7 |
|  |  | *Crocidura viaria* | 3 |
| Belenikegny | 5–6 January 2010 | *Mastomys natalensis* | 14 |
|  |  | *Mastomys erythroleucus* | 39 |
|  |  | *Mastomys huberti* | 36 |
|  |  | *Rattus rattus* | 13 |
|  |  | *Crocidura olivieri* | 9 |
| Soromba | 8–9 January 2010 | *Mastomys natalensis* | 21 |
| Komina | 8–9 January 2010 | *Mastomys natalensis* | 12 |
| Belenikegny | 3–5 September 2010 | *Mastomys natalensis* | 27 |
|  |  | *Mastomys erythroleucus* | 1 |
|  |  | *Mastomys huberti* | 16 |
|  |  | *Arvicanthis niloticus* | 1 |
|  |  | *Rattus rattus* | 9 |
|  |  | *Taterillus gracilis* | 1 |
|  |  | *Crocidura olivieri* | 5 |
| Kalibombo | 23 April 2011 | *Mastomys natalensis* | 14 |
|  |  | *Praomys daltoni* | 1 |
|  |  | *Crocidura olivieri* | 4 |
| Doucombo | 24–25 April 2011 | *Mastomys natalensis* | 24 |
|  |  | *Praomys daltoni* | 1 |
|  |  | *Acomys airensis* | 1 |
|  |  | *Crocidura olivieri* | 7 |
| Doneguebougou | 24–26 September 2011 | *Mastomys natalensis* | 41 |
| Doucombo | 30 September and 2 October 2011 | *Mastomys natalensis* | 46 |
|  |  | *Praomys daltoni* | 5 |
| Kalibombo | 1 October 2011 | *Mastomys natalensis* | 33 |
|  |  | *Praomys daltoni* | 2 |
| Kerekoumala | 5 October 2011 | *Mastomys natalensis* | 26 |
| Garalo | 5–6 October 2011 | *Mastomys natalensis* | 22 |
| Kotié | 6 October 2011 | *Mastomys natalensis* | 11 |
| **Total** |  |  | **744** |

These data were listed previously for a study on tick-borne relapsing fever in Mali [7].
