# Supplementary material for: Fleas and trypanosomes of peridomestic small mammals in sub-Saharan Mali
Source: Parasit Vectors. 2016 Oct 11;9:541. doi: 10.1186/s13071-016-1818-5 (PMC5057378; doi:10.1186/s13071-016-1818-5)
Supplement: Additional file 2: Table S2. — Date, location, host species, animal identification number, flea species, and number and sex collected. (DOC 125 kb) [file 13071_2016_1818_MOESM2_ESM.doc]

**Additional file 2: Table S2.** Date, location, host species, animal identification number, flea species, and number and sex collected

| **Date** | **Village** | **Host species** | **Animal ID No.** | | **Flea Species** | | **No. and Sex** | |
| --- | --- | --- | --- | --- | --- | --- | --- | --- |
| 12/6/07 | Molibana | *Arvicanthis niloticus* | 3 | *X. nubica* | | 1F | |  |
| 12/6/07 | Molibana | *Arvicanthis niloticus* | 9 | *X. cheopis* | | 1M | |  |
| 12/6/07 | Molibana | *Mastomys erythroleucus* | 10 | *X. nubica* | | 1M | |  |
| 12/6/07 | Molibana | *Arvicanthis niloticus* | 12 | *X. cheopis* | | 1F | |  |
| 12/6/07 | Molibana | *Arvicanthis niloticus* | 13 | *X. cheopis* | | 1M, 1F | |  |
| 12/6/07 | Molibana | *Mastomys erythroleucus* | 14 | *X. cheopis*/*nubica* | | 2M | |  |
| 12/6/07 | Molibana | *Taterillus gracilis* | 16 | *X. nubica* | | 1F | |  |
| 12/6/07 | Molibana | *Mastomys erythroleucus* | 17 | *X. cheopis* | | 1M | |  |
| 12/6/07 | Molibana | *Praomys daltoni* | 18 | *X. cheopis* | | 2M | |  |
| 12/6/07 | Molibana | *Mastomys erythroleucus* | 19 | *X. nubica* | | 1F | |  |
| 12/7/07 | Sama | *Taterillus gracilis* | 27 | *X. nubica* | | 3F | |  |
| 12/7/07 | Sama | *Taterillus gracilis* | 28 | *X. nubica* | | 1M | |  |
| 12/10/07 | Djougounte | *Crocidura olivieri* | 38 | *X. cheopis* | | 1M | |  |
| 12/10/07 | Djougounte | *Praomys daltoni* | 42 | *X. cheopis* | | 2F | |  |
| 12/11/07 | Djidian | *Mastomys natalensis* | 57 | *X. cheopis* | | 1M | |  |
| 12/11/07 | Djidian | *Mastomys natalensis* | 58 | *X. cheopis* | | 2M | |  |
| 12/11/07 | Djidian | *Mastomys natalensis* | 59 | *X. cheopis* | | 2M, 2F | |  |
| 12/11/07 | Djidian | *Mastomys natalensis* | 61 | *X. cheopis* | | 1F | |  |
| 12/11/07 | Djidian | *Mastomys natalensis* | 62 | *X. cheopis* | | 6M, 6F | |  |
| 12/11/07 | Djidian | *Mastomys natalensis* | 63 | *X. cheopis* | | 1M, 1F | |  |
| 12/11/07 | Djidian | *Mastomys natalensis* | 64 | *X. cheopis* | | 2M | |  |
| 12/11/07 | Djidian | *Mastomys natalensis* | 65 | *X. cheopis* | | 1F | |  |
| 1/12/09 | Bozokin | *Mastomys natalensis* | 68 | *X. cheopis* | | 3M | |  |
| 1/12/09 | Bozokin | *Mastomys natalensis* | 72 | *X. cheopis* | | 2M | |  |
| 1/12/09 | Bozokin | *Mastomys natalensis* | 75 | *X. cheopis* | | 4M | |  |
| 1/12/09 | Bozokin | *Mastomys natalensis* | 84 | *X. cheopis* | | 2F | |  |
| 1/13/09 | Kenieroba | *Crocidura olivieri* | 97 | *X. cheopis* | | 1M, 3F | |  |
| 1/13/09 | Kenieroba | *Mastomys natalensis* | 98 | *X. nubica* | | 1F | |  |
| 1/13/09 | Kenieroba | *Crocidura olivieri* | 99 | *X. cheopis* | | 8M, 2F | |  |
| 1/13/09 | Kenieroba | *Crocidura olivieri* | 100 | *X. cheopis* | | 3M, 2F | |  |
| 1/13/09 | Kenieroba | *Mastomys natalensis* | 104 | *X. cheopis* | | 1M | |  |
| 1/13/09 | Kenieroba | *Mastomys natalensis* | 106 | *X. cheopis* | | 1M | |  |
| 1/13/09 | Kenieroba | *Crocidura olivieri* | 107 | *X. cheopis* | | 3M, 3F | |  |
| 1/13/09 | Kenieroba | *Crocidura olivieri* | 109 | *X. cheopis* | | 2F | |  |
| 1/13/09 | Kenieroba | *Mastomys natalensis* | 110 | *X. cheopis* | | 1M, 2F | |  |
| 1/14/09 | Fourda | *Crocidura olivieri* | 111 | *X. cheopis* | | 1F | |  |
| 1/14/09 | Fourda | *Mastomys natalensis* | 118 | *X. cheopis* | | 1F | |  |
| 1/14/09 | Fourda | *Mastomys natalensis* | 119 | *X. cheopis* | | 3F | |  |
| 1/14/09 | Fourda | *Mastomys natalensis* | 122 | *X. cheopis* | | 1M | |  |
| 1/14/09 | Fourda | *Mastomys natalensis* | 124 | *X. cheopis* | | 2F | |  |
| 1/17/09 | Sinkerma | *Mastomys natalensis* | 134 | *X. nubica* | | 1M, 8F | |  |
| 1/17/09 | Sinkerma | *Praomys daltoni* | 136 | *X. nubica* | | 1F | |  |
| 1/18/09 | Petaka | *Mastomys erythroleucus* | 146 | *X. nubica* | | 1F | |  |
| 1/18/09 | Petaka | *Taterillus gracilis* | 157 | *X. nubica* | | 1F | |  |
| 1/18/09 | Petaka | *Praomys daltoni* | 164 | *X. nubica* | | 1M, 2F | |  |
| 1/18/09 | Petaka | *Mastomys natalensis* | 168 | *X. nubica* | | 1M, 1F | |  |
| 1/18/09 | Petaka | *Mastomys natalensis* | 172 | *X. nubica* | | 2M, 5F | |  |
| 1/18/09 | Petaka | *Mastomys natalensis* | 180 | *X. nubica* | | 2F | |  |
| 1/18/09 | Petaka | *Praomys daltoni* | 181 | *X. nubica* | | 1F | |  |
| 1/19/09 | Belenikegny | *Mastomys erythroleucus* | 197 | *X. cheopis* | | 1M | |  |
| 6/8/09 | Soromba | *Mastomys natalensis* | 240 | *X. cheopis* | | 1M, 1F | |  |
| 6/8/09 | Soromba | *Mastomys natalensis* | 246 | *X. cheopis* | | 1M, 4F | |  |
| 6/8/09 | Soromba | *Mastomys natalensis* | 247 | *X. cheopis* | | 1F | |  |
| 9/3/10 | Belenikegny | *Taterillus gracilis* | 457 | *X. nubica* | | 2M, 1F | |  |
| 9/3/10 | Belenikegny | *Mastomys natalensis* | 460 | *X. cheopis* | | 1F | |  |
| 9/3/10 | Belenikegny | *Mastomys natalensis* | 461 | *X. cheopis* | | 3M, 5F | |  |
| 9/3/10 | Belenikegny | *Mastomys natalensis* | 462 | *X. cheopis* | | 1F | |  |
| 9/3/10 | Belenikegny | *Mastomys natalensis* | 474 | *X. cheopis* | | 1F | |  |
| 9/3/10 | Belenikegny | *Rattus rattus* | 496 | *X. cheopis* | | 2M, 2F | |  |
| 9/3/10 | Belenikegny | *Crocidura olivieri* | 511 | *X. cheopis* | | 2F | |  |
| 9/3/10 | Belenikegny | *Rattus rattus* | 514 | *X. cheopis* | | 1M, 4F | |  |
| 4/23/11 | Kalibombo | *Crocidura olivieri* | 517 | *X. nubica* | | 1F | |  |
| 4/23/11 | Kalibombo | *Crocidura olivieri* | 519 | *X. nubica* | | 1F | |  |
| 4/23/11 | Kalibombo | *Mastomys natalensis* | 523 | *X. nubica* | | 1F | |  |
| 4/23/11 | Kalibombo | *Mastomys natalensis* | 529 | *X. nubica* | | 1F | |  |
| 4/24/11 | Doucombo | *Crocidura olivieri* | 547 | *X. nubica* | | 1M | |  |
| 9/25/11 | Doneguebougou | *Mastomys natalensis* | 589 | *X. cheopis* | | 2M, 10F | |  |
| 9/25/11 | Doneguebougou | *Mastomys natalensis* | 592 | *X. cheopis* | | 1M | |  |

*Abbreviations*: M, male; F, female
